# The 5-factor modified frailty index as a prognostic factor for stereotactic radiosurgery in meningioma management

**DOI:** 10.1007/s11060-024-04873-2

**Published:** 2024-11-14

**Authors:** Sanjeev Herr, Trent Kite, Praveer Vyas, Stephen Karlovits, Alexander Yu, Rodney E. Wegner, Matthew J. Shepard

**Affiliations:** 1 Drexel University College of Medicine, Philadelphia, PA USA; 2 Department of Neurosurgery, Allegheny Health Network Neuroscience Institute, Pittsburgh, PA USA; 3 Division of Radiation Oncology, Allegheny Health Network Cancer Institute, Pittsburgh, PA USA

**Keywords:** Frailty, Stereotactic Radiosurgery, Meningioma, mFI-5

## Abstract

**Purpose:**

Meningiomas are the most frequent primary intracranial malignancy. While surgical resection can confer long term tumor control, stereotactic radiosurgery (SRS) is often used for small, asymptomatic tumors in the adjuvant setting. Frailty has been associated with increased rates of peri-operative morbidity but has yet to be defined in the setting of SRS for meningiomas. We therefore sought to examine the relationship between frailty and clinical/radiographic outcomes of patients with meningiomas who have undergone SRS.

**Methods:**

A single-center, retrospective cohort study classified patients by their 5-factor modified frailty index (mFI-5) score into pre-frail (0–1) and frail (2–5) at the time of SRS treatment. Evaluations of overall survival (OS), progression free survival (PFS), local control (LC), and distant control (DC) were performed using Kaplan–Meier analysis. Cox proportional hazards regression analysis was used to further define factors associated with OS/PFS.

**Results:**

94 patients met inclusion criteria and underwent SRS for meningioma treatment from 2019 to 2023. Analyses compared prefrail (0–1) and frail (2–5) individuals. Kaplan–Meier analysis demonstrated a near significant association between frailty and OS (HR 3.66, 95% CI 0.49–26.8 p = 0.05) with 3-year OS rates of 75.4% in the pre-frail versus 36.6% in the frail group. However, a significant relationship was demonstrated between frailty and PFS (HR: 2.95 95% CI 1.12–7.81, p = 0.02) with 3-year PFS rates of 90.5% in the pre-frail group versus 49.2% in the frail group. Univariable regression analysis demonstrated that frailty, prior surgical excision, and cumulative tumor volume predicted PFS.

**Conclusion:**

Frailty, as assessed by the mFI-5, did not independently predict OS but did predict PFS in individuals with meningioma undergoing SRS.

## Introduction

Meningiomas, with an incidence rate of 7.86 cases per 100,000 people per year, have been identified as the most common primary intracranial tumor [1]. They are further classified into WHO grades I, II, and III based on certain histological features, brain invasion and mitotic rate [2]. The CBTRUS Statistical Report from 2012 to 2016 reported that the incidence of WHO Grade I, II, and III meningiomas were 80.5%, 17.7%, and 1.7%, respectively [3]. The 10-year survival rates of patients with meningioma are 96.8% (WHO grade I), 90.2% (WHO grade II) and 30.4% (WHO grade III) [4].

Surgical excision is often employed for large, symptomatic tumors or higher grade meningiomas [5]. Radiotherapy, specifically stereotactic radiosurgery (SRS) has been increasingly used to treat small, asymptomatic meningiomas and is often utilized in the adjuvant setting, especially for high grade meningiomas [6].

Increasing frailty has been identified as an independent, poor prognostic risk factor for patients with central nervous system (CNS) tumors. Increasing frailty has been shown to be associated with an increase in mortality and postoperative complications [7]. Two widely utilized metrics are the Charlson Comorbidity Index (CCI) and the 11-factor modified frailty index (mFI-11), which effectively have identified patients as frail, or at high risk, during surgical intervention. Although effective, these tools are not practical in the clinical setting [8, 19]. To provide a more practical tool a simplified 5-factor modified frailty index (mFI-5), as described in Table 1, was developed. An increase in mFI-5 has demonstrated an increased likelihood of postoperative mortality and complications in patients with brain metastases and gliomas [10, 11].

**Table 1:** Patient Characteristics

	Variable	Pre-frail (*n* = 79)	Frail or (*n* = 14)	p
Frailty	mFI-5	0 (0–1)	2 (2–2)	N/A
Patient characteristics	Age (years)	64.0 ± 12.3	67.1 ± 10.2	0.38
	Female Sex	57 (72.2%)	10 (71.0%)	0.99
	Body Mass Index (kg/m^2^)	30.6 ± 7.0	32.1 ± 7.0	0.44
	Race			
	1. White	74 (93.7%)	12 (85.7%)	0.28
	2. Black	3 (3.8%)	2 (14.3%)	
	3. Asian	2 (2.5%)	0 (0.0%)	
	Hispanic or Latino Ethnicity	1 (1.3%)	0 (0.0%)	0.99
	KPS > = 90	61 (77.2%)	8 (57.1%)	0.18
mFI-5 components	Hypertension	32 (40.5%)	13 (92.9%)	0.0009*
	COPD	2 (2.5%)	2 (14.3%)	0.11
	Diabetes	2 (2.5%)	11 (78.6%)	< 0.0001*
	CHF	0 (0.0%)	2 (14.3%)	0.021*
	Need Assistance	0 (0.0%)	2 (14.3%)	0.021*
Prior treatment	Surgical Excision	43 (54.4%)	10 (71.4%)	0.37
	Gamma Knife	3 (3.8%)	1 (7.1%)	0.49
	External Beam Radiation	10 (12.7%)	4 (28.6%)	0.21
	Medical Management	0 (0.0%)	0 (0.0%)	0.99
Follow-up	Clinical (months)	16 (10–27)	13 (10–24)	0.61
	Radiographic (months)	15 (9–27)	11 (7–20)	0.30

Quantitative data presented as mean ± standard deviation or median (interquartile range)

Categorical data presented as count (percentage)

*p < 0.05 significant

*CHF* congestive heart failure, *COPD* chronic obstructive pulmonary disease, *KPS* Karnofsky performance status, *mFI-5* 5-factor modified frailty index

Frailty has not been adequately studied in patients undergoing SRS with meningiomas. As SRS is often utilized in patients who have undergone prior surgical excision or who are at increased risk for complications following craniotomy, understanding the impact of patient frailty on radiosurgical outcomes for patients with meningiomas is imperative. As a result, we sought to study the relationship between frailty and tumor control, as well as overall survival, in patients with WHO grade I, II and III meningiomas undergoing SRS.

## Methods

A single-center retrospective cohort study was performed at Allegheny General Hospital in Pittsburgh, Pennsylvania. Patients with a diagnosis of meningioma receiving SRS between May 2019 and May 2023 were identified using an institutional database. Institutional review board approval was obtained.

Patients were treated with frame or mask-based SRS using the Gamma Knife Icon Unit (Elekta, Stockholm, Sweden), 1–5 fractions were utilized, and dose selection was determined by the treating neurosurgeon, radiation oncologist and radiation physicist.

A retrospective review of radiographic and clinical data obtained manually from patient electronic health records was then conducted. Baseline demographics including patient age, sex, meningioma location, WHO classification and cancer treatment history were assessed. An mFI-5 score of 0–5 was determined by assigning a point for each of the following variables: history of diabetes mellitus, hypertension, chronic obstructive pulmonary disease, congestive heart failure and need for assistance in activities of daily living (Karnofsky performance status (KPS) ≤ 60) prior to SRS treatment.

Given the power of our study, we aggregated the results of frail patients (mFI-5 2–3) and severely frail (mFI-5 3 +) and compared that group against pre-frail patients (mFI-5 0–1) for our final analysis. Quantitative variables were analyzed using the two-sample *t*-test or Wilcoxon rank sum test and are presented as mean ± standard deviation or median (interquartile range). Categorical variables were analyzed using a two-way Fisher’s exact test and are reported as count (percentage). Variables with 3 groups were analyzed using a three-way Fischer’s exact test and are reported as count (percentage). Overall survival (OS), progression-free survival (PFS), local control (LC) and distant control (DC) were analyzed using a Kaplan–Meier survival analysis OS was defined as time from initiation of treatment of the index lesion to death. PFS was defined as time from initiation of treatment until tumor progression or death, whichever came first [12–17] Total control was defined as a function of local and distant progression. Survival probabilities are reported with 95% confidence intervals (CIs).

Univariable Cox proportional-hazards regression was performed to determine whether frailty (based on mFI-5), age, sex, prior surgical excision, interval from surgery to radiosurgery and cumulative tumor volume were associated with OS and PFS. To obtain a potentially less-biased estimate for the association between frailty category and both OS and PFS, bivariable models with the covariates of frailty category and each variable that almost approached significance (p < 0.10) in the univariable model were fit to the data. Hazard ratios (HRs) are reported with 95% CIs.

A significance level α of 0.05 was used for all tests. Kaplan Meier plots were constructed using GraphPad Prism version 10.3.1. All other analyses were performed using R version 4.2.1.

## Results

Ninety-three patients met inclusion criteria and were evaluated in this retrospective review, with clinical and demographic characteristics presented in Table 1. The mean age of patients in the pre-frail group was 64.0 ± 12.3 with 57 (72%) being females and 22 (28%) being males. The mean age of patients in the frail group was 67.1 ± 10.2 with 10 (71%) being females and 4 (29%) being males. The median radiographic follow up was 15 months (IQR: 9–27) in the pre-frail group compared to 11 months (IQR: 7–20) in the frail group (p = 0.30). The median clinical follow up was 16 months (IQR: 10–27) in the pre-frail group compared to 13 months (IQR: 10–24) in the frail group (p = 0.61). Most patients were classified as prefrail (79, 84.9%) and the remainder classified as a group of frail/severely frail (14, 15.1%). No significant difference was observed between the two groups regarding age, sex, BMI or race.

As expected, patients in the frail cohort had increased rates of hypertension, diabetes, heart failure and the need for assistance with daily living (Table 1). The rates of COPD were similar in both cohorts.

Most patients in both groups had undergone surgical excision prior to SRS with a notably higher proportion in the frail/severely frail group (10, 74.1%) compared to the pre-frail group (43, 54.4%), although this was not found to be significant. The history+ of prior external beam whole brain radiation was less common but still more frequent in the frail/severely frail group (4, 28.6%) versus the pre-frail group (10, 12.7%), although this was also not found to be significant.

**Table 2:** Tumor and Radiosurgery Information

Variable	Pre-frail (n = 79)	Frail (n = 14)	p
Tumor location			
1*.* Cavernous	3 (3.8%)	0 (0.0%)	0.61
2. Cerebellopontine angle	4 (5.1%)	0 (0.0%)	
3. Convexity	15 (19.0%)	1 (7.1%)	
4. Intraventricular	1 (1.3%)	0 (0.0%)	
5. Parafalcine	17 (21.5%)	6 (42.9%)	
6. Parasagittal	12 (15.2%)	1 (7.1%)	
7. Skull base	27 (34.2%)	6 (42.9%)	
Cumulative tumor volume (cm^3^)	6.3 ± 7.3	12.1 ± 13.5	0.021***
WHO classification*			
1. Grade 1	23/43 (53.5%)	3/9 (33.3%)	0.47
2. Grade 2	20/43 (46.5%)	6/9 (66.7%)	
3. Grade 3	0/43 (0.0%)	0/9 (0.0%)	
Interval between prior surgery and SRS (months)**	57.9 ± 71.9	32.4 ± 46.2	0.29
Tumor resection cavity treatment only	32 (43.2%)	8 (61.5%)	0.36
Number of tumors treated	1 (1–1)	1 (1–1)	0.88
Fractions of SRS	5 (3–5)	5 (3–5)	0.86
Margin dose (Gy)	23.4 ± 5.6	25.0 ± 5.2	0.31
Maximal dose (Gy)	46.6 ± 11.3	50.1 ± 10.5	0.28

Quantitative data presented as mean ± standard deviation or median (interquartile range)

Categorical data presented as count (percentage)

*Of the 52 patients for whom histopathologic analysis was available

**Of the 52 patients who underwent surgical intervention

***p < 0.05 significant

*SRS* stereotactic radiosurgery, *WHO* World Health Organization

Within this cohort of patients, pathology was available for 52 patients (55.9%) and pathology revealed 26 patients with WHO grade I meningiomas and 26 with WHO grade II meningiomas (Table 2). Pre-frail patients had a smaller proportion of confirmed higher grade meningiomas with 23 patients (53.5%) having WHO grade I meningiomas and 20 patients (46.5%) having WHO grade II meningiomas. The frail group demonstrated a predilection towards higher grade meningiomas with 66.7% (n = 6) confirmed as WHO grade II and 33.3% (n = 3) confirmed WHO grade I, although this was not significant. The frail group also demonstrated a significantly (p = 0.021) greater tumor volume (12.1 ± 13.5) vs. the pre-frail group (6.3 ± 7.3). Radiosurgical variables were similar across both cohorts (Table 2).

**Fig. 1:**
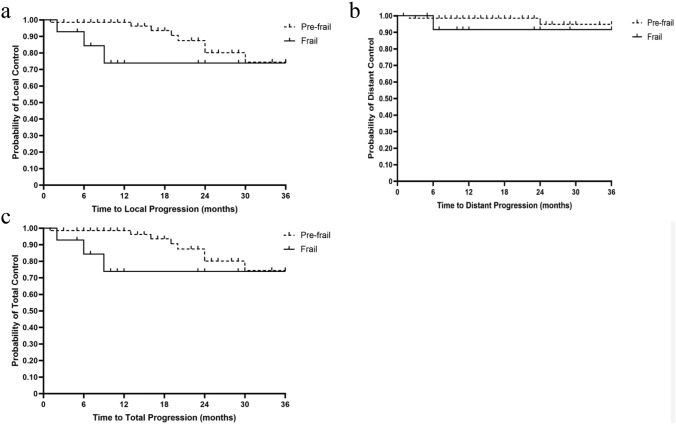
**a** Kaplan–Meier plot of local control for all patients at 36 months. **b** Kaplan–Meier plot of distant control at 36 months. **c** Kaplan–Meier plot of total control 36 months stratified by frailty status

Kaplan–Meier analysis was then performed to examine probabilities of local, distant and total control. The 1-,2-, and 3-year probability of local control in the pre-frail group was 98.6%, 80.2% and 74.4% respectively. The 1-,2-, and 3-year probability of local control in the frail group was 73.9%, 73.9%, and 73.9% respectively (Fig. 1a). This demonstrated a significant difference between the pre-frail and frail groups with respect to local control (HR 0.22, 95% CI 0.036–1.42, p = 0.01). The 1-, 2-, and 3-year DC probability for pre-frail patients was 98.5%, 94.9%, and 94.9% respectively, while the 1-,2-, and 3-year DC probability for frail patients was 91.6%, 91.6%, and 91.6% respectively (Fig. 1b). This relationship demonstrated no significant difference between the pre-frail and frail group with respect to distant control (HR 0.26, 95% CI 0.01–5.30, p = 0.26). The 1-,2-, and 3-year probability of total control in the pre-frail group was 98.6%, 80.2%, and 74.4% respectively. The 1-, 2-, and 3-year probability of total control was 73.9%, 73.9%, and 73.9% respectively (Fig. 1c).

**Fig. 2:**
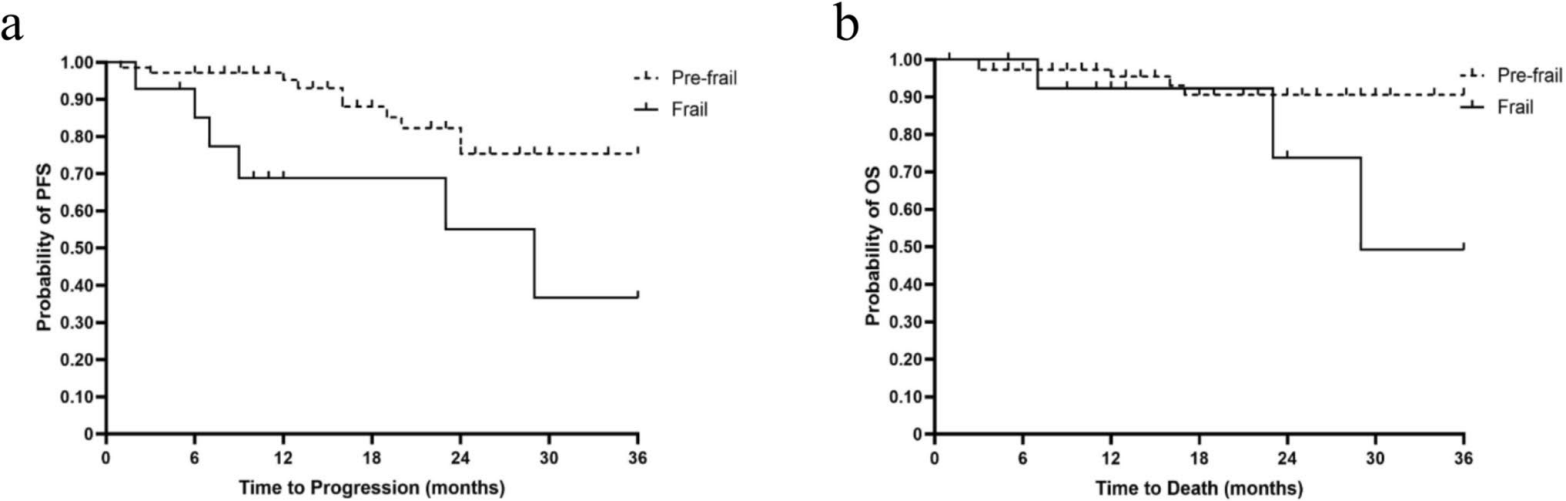
**a** Kaplan–Meier plot of progression free survival at 36 months stratified by frailty status. **b** Kaplan–Meier plot of overall survival at 36 months stratified by frailty status

Kaplan–Meier analysis also demonstrated a significant association (p = 0.02) between frailty and PFS (Fig. 2a). The 1-,2-, and 3-year PFS probability for pre-frail patients was 95.2%, 75.4%, 75.4% respectively. The 1-,2-, and 3-year PFS probability for frail patients was 68.7%, 55.0%, and 36.6% respectively. There was a trend towards diminished OS with increasing frailty after SRS for meningiomas although this did not achieve statistical significance (p = 0.05). The 1-, 2-, and 3-year OS probability for pre-frail patients was 95.5%, 90.5%, and 90.5% respectively. The 1-, 2-, and 3-year OS probability in the frail group was 92.3%, 73.8%, and 49.2% respectively (Fig. 2b).

On univariate analysis (Table 3) there was an association between frailty and progression free survival (PFS) (HR: 2.95 (1.09–7.59); p = 0.033). Prior surgical excision, interval between surgery and radiosurgery and cumulative tumor volume were all associated with earlier tumor progression. In a bivariable model adjusting for interval to radiosurgery, frailty maintained an association with PFS (HR for frailty: 3.45 (1.07–11.07); p = 0.038). Of note, prior surgical excision may represent a clinically significant risk factor of progression.

**Table 3:** Univariable regression analysis of overall survival and progression-free survival

Predictor	Hazard ratio (95% CI)	p
Overall survival		
Frail status (mFI-5 ≥ 2)	3.68 (0.88–15.45)	0.075
Age (years)	1.06 (1.00–1.13)	0.060
Female sex	3.21 (0.39–26.10)	0.28
Prior surgical excision	2.38 (0.48–11.82)	0.28
Interval between prior surgery and radiosurgery (months)	1.01 (1.00–1.02)	0.044*
Cumulative tumor volume (cm^3^)	1.03 (0.97–1.10)	0.30
WHO classification		
1. Grade 1	(Reference)	0.37
2. Grade 2	1.93 (0.46–8.21)	
Progression free survival		
Frail status (mFI-5 ≥ 2)	2.95 (1.12–7.81)	0.029*
Age (years)	1.02 (0.97–1.06)	0.46
Female sex	1.00 (0.38–2.63)	0.99
Prior surgical excision	3.27 (1.08–9.86)	0.035*
Interval between prior surgery and radiosurgery (months)	1.01 (1.00–1.01)	0.049*
Cumulative tumor volume (cm^3^)	1.05 (1.01–1.08)	0.015*
WHO classification		
1. Grade 1	(Reference)	0.0018*
2. Grade 2	4.27 (1.71–10.64)	

*p < 0.05 significant

*CI* confidence interval, *mFI-5* 5-factor modified frailty index, *WHO* World Health Organization

## Discussion

This is the only study examining outcomes in meningioma patients stratified by mFI-5. The results herein suggest that frail patients tend to have decreased PFS and a trend towards diminished OS. We also identified local control as the primary driver of total control. This is consistent with the nature of meningiomas in that they are highly locally recurrent, especially in the context of inadequate tumor resection or SRS volume coverage. SRS in the setting of meningiomas has been established as an efficacious management strategy in a variety of grades and clinical settings. Appropriate patient selection and risk stratification can better inform the timing and ultimately the decision to proceed with therapy.

We have previously examined the influence of frailty, as measured by the mFI-5, in patients undergoing SRS for brain metastases [18]. Previously it was demonstrated that increasing frailty scores were associated with diminished OS and decreased intracranial PFS, which was further corroborated by this study.

It is rational to assume that there is a relationship between increasing frailty and a trend towards diminished OS. However, the direct influence of frailty on intracranial disease control is less clear. In the context of brain metastases, we have previously postulated that increasing frailty may be associated with poor tolerance to systemic chemotherapy, immunotherapy or targeted therapy and as a result earlier intracranial disease progression. [19]

The interplay between frailty and meningioma progression is not understood. In this study, frailty tended to be observed in patients with higher grade tumors and more aggressive prior treatment and thus we cannot exclude that frailty is a confounder here. Notably, however, there was no statistical difference in the incidence of high grade meningiomas in the frail and pre-frail cohorts. Furthermore, many patients without prior surgery were included in this analysis. Our study is underpowered for multivariate analysis and further work will be needed to refine these findings. Nevertheless, it has been previously recognized that increasing frailty is associated with aberrations in baseline levels of inflammation and dysregulation of the adaptive immune response [20]. Thus, there may be a biologic basis supporting increased frailty status and the association with earlier tumor progression.

Post-radiosurgical parenchyma releases a variety of cytokines to recruit monocyte/macrophage cell types to engage in wound healing, tissue debris trafficking, and neo-angiogenesis [21]. The earlier tumor progression seen in frailer patients may be a product of a less robust post-radiosurgical immune response enabling further tumor growth.

One question at hand is whether frailty can be modified to optimize surgical and possibly radiosurgical outcomes. The concept of pre-habilitation has been explored in surgical oncology and gynecologic oncology patients. Patients enrolled in aggressive physical therapy and nutritional optimization prior to surgical intervention have improved surgical outcomes and decreased perioperative morbidity [22, 23]. Likewise, introducing an exercise regimen in patients with breast cancer led to an upregulation of several gene expression pathways involved in immunity and inflammation [24]. Whether a multimodal enhanced recovery pathway has any role in improving patient outcomes in the radiosurgical space is worth exploring, especially in patients who are frail or who have higher grade meningiomas.

Increased tumor volume in meningioma has been associated with higher Ki-67 index and is thought to harbor cell subsets with intrinsic radio resistance and proliferative properties [25]. Thus, it is not surprising that patients with larger tumors on initial presentation tend to undergo local failure with resultant recurrence. Additionally, patients with larger tumors are less likely to receive SRS and more likely to undergo resection alone and therefore have greater probability of residual disease, potentially explaining the relationship between surgical resection and diminished PFS. Furthermore, univariate analysis exhibited a negative significant relationship between time from diagnosis to SRS on PFS. Delaying therapy may result in a larger treatment volume challenging resection or complete target volume coverage with SRS. Our results corroborated the effect of time from diagnosis to SRS as a negative survival factor [26].

This study provides important insights into the potential role of frailty in influencing outcomes for patients with meningiomas undergoing SRS. The fundamental premise of frailty as a clinical tool is the ability to quantitatively estimate an individual's fitness for surgery/adjuvant treatment through the use of parameters agnostic to the underlying tumor pathology. The frailty index has the potential to provide an evidence-based classification tool by which more robust prognostication can be achieved. Typically, patients and their fitness to undergo relatively aggressive therapies is determined largely by clinical gestalt, and prognostic features limited to specific features of a patients pathology (for example Ki67 index in meningioma or IDH status in gliomas).

The mFI5 is able to capture simple comorbidities which are not specific to the underlying pathology, thus permitting generalizability to all patients in a variety of clinical settings and provide an estimate of a patients fitness for treatment. Understanding that increasing frailty can lead to increased adverse events while also curtailing PFS can be informative to neurosurgeons weighing the risks/benefits of aggressive surgical treatment. Our data suggests that frail patients have earlier time to meningioma progression after SRS. While this may not directly change management at this time, this is an important observation that we have previously demonstrated in a variety of disease states including patients undergoing SRS for brain metastases. It is also important to note that frailty may not be static. Whether or not a more robust, pre-habilitation program encompassing supportive care, nutrition and physical therapy can improve one’s response to SRS has never been investigated. In order to assess whether prehabilitation programs have merit in the neurosurgical oncology space, it is imperative that we have a benchmark on which to monitor progress.

As with any retrospective study, there are limitations of our study including the possibility of confounding and recall/selection bias. The relatively small sample size, particularly in the frail group, limited our ability to perform a robust multivariate analysis which in turn limited our ability to minimize potential confounders. Given the imbalance in sample sizes between the pre-frail (n = 79) and frail (n = 14) groups, we acknowledge that the study may have been underpowered to detect some differences. Very few patients met the criteria for “frail” therefore we combined both the “frail” and “severely frail” subgroups for analysis. As a result, a certain level of granularity may not have been captured while analyzing results across subgroups. Another notable limitation is the relatively short follow-up period given the typically indolent nature of meningiomas. Unfortunately, this is partly due to the timing of data collection in relation to the SRS procedure, as well as logistical challenges such as patient relocation or cessation of follow-up visits, despite hospital efforts to ensure continued monitoring. Finally, one particularly challenging confounding variable is WHO grade. Given the inherent differences in the aggressiveness between WHO grade 1 and 2 meningiomas it is a reasonable expectation that the inclusion of higher proportions of the more aggressive WHO grade 2 tumors may influence progression. While our cohort did not demonstrate a significant difference in the proportions of WHO grade 1 or 2 meningiomas across frailty groups, there was a high proportion of grade WHO grade 2 tumors in the frail group. The relative rarity of higher grade meningiomas (grades 2 and 3) will likely preclude a single institution from enrolling a sample sufficient for conducting a regression analysis accounting for the role of WHO grade across frailty groups. This highlights the need for future multi-institutional studies to provide sufficient samples necessary for such analyses. Given the limitations of this study we do acknowledge that our work is hypothesis-generating in nature and that further studies with larger sample sizes, longer follow-up and more advanced statistical analyses will be needed to expand upon these results.

## Data Availability

Data obtained from patient records and utilized for analysis and generation of results cannot be publicly shared in order to protect patient privacy.
